# Relationship between level of serum VIT D and insulin resistance in patients with MAFLD

**DOI:** 10.1186/s12902-024-01820-0

**Published:** 2025-07-14

**Authors:** Alshymaa A. Hassnine, Amr M. Elsayed, Samar K. Abdelaziez, Mahmoud M. Higazi, Nagwa I. Okaily, Wael Soliman

**Affiliations:** 1 Department of Tropical Medicine, Faculty of Medicine, Minia University, Minia, Egypt; 2 Department of Radiology, Faculty of Medicine, Minia University, Minia, Egypt; 3 Department of Clinical Pathology, Faculty of Medicine, Minia University, Minia, Egypt

**Keywords:** Metabolic associated fatty liver disease, Vitamin D, HOMA-IR, Non-alcoholic fatty liver disease, Fibrosis

## Abstract

**Background:**

Non-alcoholic fatty liver disease has been recognized as the most common metabolic liver disease in Western countries, and its prevalence is increasing all over the world. Metabolic syndrome and diabetes mellitus (DM) are closely linked to non-alcoholic fatty liver disease. Because of the close relationship between fatty liver and metabolic illnesses, NAFLD has lately been renamed as Metabolic Associated Fatty Liver Disease. HOMA-IR is thought to be the best indicator of the progression of fatty liver to advanced cirrhosis and fibrosis.

**Aim:**

to assess the relation between level of serum VIT D and insulin resistance in patients with MAFLD.

**Patients & methods:**

This cross sectional observational study included 120 subjects (60 control & 60 MAFLD patients), where clinical examination including anthropometric measurements, laboratory tests (CBC, liver and renal function, metabolic profile, serum VIT D, HOMA-IR), ultrasonography and shear wave elastography were performed on all the subjects.

**Results:**

In MAFLD patients, serum vitamin D levels were statistically significant lower (55%) than in the non-MAFLD group (15%), and they also significantly decreased as steatosis and fibrosis grades increased (*P* < 0.001). In addition, there is an inverse correlation between serum vitamin D levels and IR in MAFLD patients, but it is not statistically significant.

**Conclusion:**

Vitamin D supplementation may be helpful in the treatment of MAFLD since people with the condition have lower serum vitamin D levels than those without it, and these levels drastically decreased with increasing grades of fatty liver.

## Introduction

Non-alcoholic fatty liver disease has been recognized as the most common metabolic liver disease in Western countries, and its prevalence is increasing all over the world [1]. It is affecting 18–46% of the adults [2]. Younossi et al. reported its prevalence to be highest (32%) in the Middle East, paralleling to the prevalence of the metabolic syndrome [3]. NAFLD is also present in 7% of normal-weight individuals. It is characterized by the consequences of several hepatic disorders, ranging from simple hepatic steatosis to steatohepatitis and fibrosis, liver cirrhosis and even HCC [3, 4]. NAFLD has been associated strongly with diabetes mellitus and metabolic syndrome. The prevalence of fatty liver in diabetic patients has been reported about 70% [3, 4]. Because of the close relationship between fatty liver and metabolic illnesses, NAFLD has lately been renamed as Metabolic Associated Fatty Liver Disease. HOMA-IR (HOMA Insulin Resistance) is thought to be the best indicator of the progression of fatty liver to advanced cirrhosis and fibrosis [4]. When MAFLD has established it increases the hepatic IR, which, may be triggered towards NASH, liver cirrhosis, hepatic cell failure, and hepatocellular carcinoma, in 30–40% of cases [5]. MAFLD may encourage systemic low-grade inflammation and raise insulin resistance in extra-hepatic organs [6]. It also, considers a known risk factor for cardiovascular disease complications [7].

Low serum vitamin D levels are linked to the occurrence of complications related to insulin resistance, such as type 2 diabetes [8], metabolic diseases [9], and MAFLD [10–12]. Regarding the association between fatty liver and vitamin D, different clinical trials recently studied the efficacy of supplementation of vitamin D in MAFLD patients, with controversial results [10, 13].

Numerous studies examine how vitamin D affects glycemic control and insulin resistance in diabetic individuals with conflicting and controversial results; Our goal is to evaluate the relationship between level of serum vitamin D and insulin resistance in MAFLD patients.

## Patients & methods

Our study is a cross-sectional study, during the period from March to December 2022. This study included 120 persons who attended the outpatient clinic for fatty liver disease at Minia hospital (60 control & 60 MAFLD patients).

All subjects fulfilled the following criteria.

**Inclusion criteria**:

- aged ≥ 18 years.
- Both sexes were included.

**Exclusion criteria**:

- Diabetic patients.
- Patients with history, clinical, or laboratory findings of any other liver diseases, like viral hepatitis, alcoholic, autoimmune, metabolic liver disease, liver cirrhosis, and HCC, were all excluded.

All patients will be subjected to:

1. History taking including diet, exercise, and special habits.
2. Examination and Anthropometric measurements.

Body mass index (BMI): calculated as weight (kg) / [height (m)]^2^, normal BMI is 18.5–24.9 kg/m^2^ (248).

Waist circumference (WC): Normal diameter is men ≤ 102 cm, women ≤ 88 cm.

3. Laboratory investigations:

CBC, liver and renal function.

Metabolic profile (lipid profile, CRP, uric acid, fasting insulin, fasting glucose, HBA1c).

Serum vitamin D.

Homa insulin resistance.

Blood Sampling Protocol.

After overnight fasting, about 7 ml of venous blood were withdrawn from each subject under aseptic conditions and divided into two tubes:

1**–**2 ml of blood was evacuated in Ethylene Diamine Tetra acetic Acid (EDTA) containing tube for complete blood count (CBC) and HbA1c.

**2–**5 ml of blood in plain tube, left until clotted and then centrifuged for obtaining serum, which was used for assaying other investigations as: fasting blood glucose level, renal function tests (blood urea and serum creatinine), liver function tests including (total bilirubin, direct bilirubin, Alanine transaminase (ALT), Aspartate transaminase (AST), and albumin) and complete lipid profile. The remaining serum was preserved at – 20 ºC for assaying of: fasting insulin level, serum vitamin D level, and C-reactive protein titre (CRP titre).

Laboratory investigations:

- CBC: Which was performed using 5-part diff Celltac G, NIHON KOHDEN CORPORATION, AUTOMATED HEMATOLOGY ANALYSER, Japan.
- Fasting blood glucose, Renal function tests (blood urea and serum creatinine) and liver function tests (total bilirubin, direct bilirubin, Alanine transaminase (ALT), Aspartate transaminase (AST), and albumin) were performed using the auto-analyzer SELECTRA PRO XL, ELITech Group, clinical chemistry automation systems, Netherlands, using the commercially available kits according to the manufacturer’s instructions.
- Complete lipid profile, including total cholesterol, triglycerides, high- density lipoprotein (HDL), Low-density lipoprotein (LDL) were done using fully automated clinical chemistry auto-analyzer system Konelab 20i (Thermo-Electron Incorporation, Finland).
- CRP titre: Using GENRUI, biotech Inc, Kinetic Assay, China.
- Glycated hemoglobin (HbA1c): by (NycoCard READER II, Finland).
- Fasting insulin: by ELISA (bioactiva diagnostic GmbH, Germany) (catalog No. BDIN31-BA).The optical density was determined using a microplate reader (Huma reader 3700, Germany).
- VIT D: by ELISA (ZEUS Diagnostic Inc., USA), (REF: ZSVTD501- DG). The optical density was determined using a microplate reader (Huma reader HS 3700, Germany).
- Homa insulin resistance:

HOMA-IR = (Fasing insulin (mU/L) × Fasting glucose mg/dL) /405.

for glycemia in mg/dL and insulin is in mU/L, (insulin resistance was defined as HOMA-IR > 1.6 in male and > 1.9 in female)

4. Radiology:
  - Abdominal u/s: using a high-resolution B-mode ultrasound with a 3.5-MHz.

-The following findings were assessed: **(1)** diffuse increased echogenicity of the liver relative to the right kidney; **(2)** attenuation of the ultrasound beam; **(3)** poor visualization of the intra-hepatic vasculature wall and architectural details.

b. Two-dimensional shear wave elastography was done to determine the degree of fibrosis in hepatic tissues. The same ultrasound system and convex probe was used for 2D SWE. The transducer was placed inter-costally at right lobe of the liver, with the target area located in the right anterior hepatic segment at an ideal depth of 3 cm to 5 cm from liver capsule (at least 1 cm depth), the size of the 2D-SWE sample box was about 3 × 3 cm. If possible, the patient should hold breath for 5–10 s. So-called Elastography Propagation Maps were acquired, which indicate the derived liver stiffness in colour-coded form.

Version 24 of the Statistical Package for Social Sciences program (SPSS) was used to enter, tabulate, and statistically analyze the obtained data. Quantitative data were expressed as mean + standard deviation, whereas qualitative data were expressed as proportions. P values less than 0.05 were considered statistically significant.

## Result

All patients (120) divided into 2 groups:

1. Non MAFLD group included 60 patients, 51 females, and 9 males (control).
2. MAFLD group included 6o patients, 17 males and 43 females.

Table 1 show the socio-demographic data and antheropometric measures of the studied subjects, there is significant increase in body weight, BMI and waist circumference in MAFLD patients than in control with p value ) *< 0.001*) respectively, there is increase in fatty diet in MAFLD patients than non- MAFLD with significant p value (*0.007).* Table 2 show laboratory investigations between the control and MAFLD groups, HOMA-IR is significant higher in MAFLD patients than non- MAFLD, there is a significant difference between the two groups in vitamin D serum level, hypovitaminosis D is present in the two groups, it is significantly increased in MAFLD group in comparison with non MAFLD. Table 3 shows fibrosis scores and steatosis staging between the control and MAFLD group. Table 4 shows relation between serum vitamin D, fibrosis scores, and steatosis staging in the studied groups. The data show that an increasing degree of steatosis and fibrosis is significantly related to vitamin D deficiency with a p value < 0.001 in MAFLD patients. Table 5 shows the correlation between vitamin D and IR in the studied groups, the result shows that vitamin D level is inversely correlated with HOMA-IR without being statistically significant in MAFLD patients. Table 6 shows logistic regression analysis for prediction of insulin resistance, the data show that hypovitaminosis D, increased age, WC, high TG, and LDH are independent factors for prediction of insulin resistance in patients with MAFLD.

## Discussion

Non-alcoholic fatty liver disease has been recognized as the most common metabolic liver disease in Western countries, and its prevalence is increasing all over the world [1]. There is documented evidences show that liver steatosis and fibrosis are established risk factors for the development of liver cirrhosis, HCC, and also, cardiovascular complications [7]. In our study, we found that there is an increase in body weight, BMI, and WC in the MAFLD group than in the control group, the same result was documented by Rocha et al., who reported the higher rates of waist circumference and BMI between NAFLD subjects and established that WC may be the best parameter for prediction of MAFLD [14], a recent study reported a substantial increase in the prevalence of MAFLD with increasing WC and BMI [15]. In our study regarding dietary habits there was no difference between 2 groups in carbohydrates and protein, but there was an increase in fatty diet in the MAFLD group than in the control, our result agrees with the study of Mirmiran et al., who reported that consumption of sweet, fat, and red meat was higher, whereas intake of vegetables, fruits, and grains was found to be less between the MAFLD subjects [16].

According to our findings, the MAFLD group had higher levels of TG, LDL, ALT, AST, and cholesterol than the control group, a significant association between abnormal lipid profile and fatty liver as cholesterol, TG, LDL, and HDL was established by Tayyem et al. [17], the same result was shown by Sathiaraj et al., who concluded that total cholesterol, TG, and low HDL were statistically significant high in patients with NAFLD [18].

According to our study, MAFLD patients are more likely than non-MAFLD patients to have hypovitaminosis D, the same results were documented by Targher et al. [19], who demonstrated that 60 patients with biopsy-proven NAFLD had lower serum levels of VIT D than patients without NAFLD., additionally, Barchetta et al. [20] reported that regardless of the presence of diabetes mellitus, serum hypovitaminosis D was associated with NAFLD identified by ultrasonography. In our study we proved that a higher VIT D deficiency is linked to a higher steatosis grading in patients with MAFLD, the same result were documented by Dasarathy et al. [21] who discovered that 148 subjects with NAFLD had significantly lower concentrations of 25(OH)D, and that these lower levels were significantly correlated to higher grades of fibrosis and steatosis in those patients, In addition, Targher et al. [19], found that low levels of 25(OH)D were closely associated with the histological severity of liver fibrosis and steatosis independent of metabolic syndrome.

In our study we found an increase in insulin resistance in MAFLD patients than in the control group, a systematic review found that NAFLD was associated with nearly two fold increase in the incidence of DM and metabolic syndrome over a period of 5 years [22], a recent study concluded that metabolic complication was frequently associated with fatty liver, including dyslipidemia, obesity, hypertension, and DM [4].

In this study we found that the serum level of vitamin D is inversely associated with HOMA-IR in the general population and MAFLD patients, the same result was reported by Barrea et al. [23], who concluded an inverse correlation between serum vitamin D and both HOMA-IR and fasting plasma insulin, these results are consistent with multiple studies [24, 25]. A possible mechanism explaining association between vitamin D metabolism and glycemic control may be due to chronic poor glycemic control which can affect metabolism of vitamin D through decreasing activity of the cytochrome P450–dependent steroid hydroxylases enzymes [26], in contrast, Leitão et al. [27] reported no correlation between insulin resistance and serum level of vitamin D, these finding is consistent with other observations of Italian studies demonstrating that serum vitamin D was inversely associated with fatty liver on ultrasound in 262 subjects independent of metabolic syndrome features including HOMA-IR [20], further, another study demonstrated a very strong inverse correlation between serum vitamin D level and fatty liver in patients with less severe NAFLD independent of confounding factors like DM [28], the mechanism by which serum VIT D deficiency can cause fatty liver independent of IR can be attributed to decrease or loss of its beneficial effects on the liver (decrease oxidative stress), on the adipose tissue (decrease its dysfunction and inflammation ) and on the gut (decrease dysbiosis and inflammation) [29].

## Conclusion

In the MAFLD population, hypovitaminosis D is common and may contribute to the occurrence of the disease. We need to do a RCT to conclude the effectiveness of vitamin D supplementation in improving MAFLD.

**Table 1:** Demographic data and diet habits between control and MAFLD group

		Control	MAFLD	*P* value
		(*N* = 60)	(*N* = 60)	
Age (year)	*Range* *Mean ± SD*	(21–29) 24.9 ± 2.5	(18–66) 40.8 ± 11.7	< 0.001*
Sex	*Male* *Female*	9(15%) 51(85%)	17(28.3%) 43(71.7%)	*0.076*
Smoking	*No* *Smoker*	60(100%) 0(0%)	57(95%) 3(5%)	*0.079*
Body Weight (kg)	*Range* *Mean ± SD*	(55–76) 64.1 ± 4.9	(65–125) 87 ± 9.7	< 0.001*
Height (cm)	*Range* *Mean ± SD*	(156–176) 164.8 ± 6.3	(150–184) 164.9 ± 7.3	*0.920*
Body Mass Index	*Range* *Mean ± SD*	(19.4–25) 23.6 ± 1.4	(25.6–44.8) 32.1 ± 4.1	< 0.001*
Waist (cm) Circumference	*Range* *Mean ± SD*	(60–98) 71.3 ± 8.8	(72–144) 106.4 ± 12.1	< 0.001*
Carbohydrates	*No* *Yes*	0(0%) 60(100%)	0(0%) 60(100%)	*----*
Protein	*No* *Yes*	0(0%) 60(100%)	1(1.7%) 59(98.3%)	*0.315*
Fat	*No* *Yes*	22(36.7%) 38(63.3%)	9(15%) 51(85%)	0.007*
Exercise	*Low* *Normal daily* *High*	0(0%) 60(100%) 0(0%)	5(8.3%) 51(85%) 4(6.7%)	0.008*
Artificial Fructose in Diet	*No* *Yes*	21(35%) 39(65%)	23(38.3%) 37(61.7%)	*0.705*

Independent Samples T test for parametric quantitative data between the two groups

Chi Square test for qualitative data between the two groups

*: Significant level at P value less than 0.05

**Table 2:** Laboratory investigations between the control and MAFLD group

		Control	MAFLD	*P* value
		*N* = 60	*N* = 60	
HBA1c	*Range* *Mean ± SD*	(4.1–5.3) 4.7 ± 0.3	(4.5–6.3) 5.4 ± 0.3	< 0.001*
Plasma Glucose (R) [mg/dL]	*Range* *Mean ± SD*	(60–100) 78.2 ± 9.7	(45–110) 78.3 ± 12.9	*0.955*
INSULIN(mU/L)	*Median* *IQR*	6.7 (3.3-8)	9.6 (5.8–16.5)	< 0.001*
HOMA IR	*Median* *IQR*	1.3 (0.8–1.5)	1.9 (1.1–3.2)	< 0.001*
Insulin resistance	*No* *Yes*	60(100%) 0(0%)	34(56.7%) 26(43.3%)	< 0.001*
Cholesterol (mg/dL)	*Range* *Mean ± SD*	(110–195) 155.5 ± 21.7	(100–356) 199.9 ± 47.8	< 0.001*
Triglyceride (mg/dL)	*Median* *IQR*	97.5 (86.3–115)	122.5 (110-157.5)	< 0.001*
HDL (mg/dL)	*Range* *Mean ± SD*	(30–50) 40 ± 5	(28–65) 38.1 ± 5.8	*0.053*
LDL(mg/dL)	*Median* *IQR*	95 (83.3–109)	120.5 (102-164.3)	< 0.001*
CRP (mg/L)	*Median* *IQR*	5.4 (4.8–6.2)	6 (4.6–16.3)	0.041*
VIT D (ng/Ml)	*Median* *IQR*	29.9 (25.5–33)	18.8 (12.5–25.6)	< 0.001*
	*Normal* *Hypovitaminosis*	51(85%) 9(15%)	27(45%) 33(55%)	< 0.001*
Hemoglobin (gm/Dl)	*Range* *Mean ± SD*	(9.7–13.9) 11.8 ± 1.1	(9.9–16.3) 13.1 ± 1.4	< 0.001*
Platelet Count	*Range* *Mean ± SD*	(168–433) 278.3 ± 58.3	(150–464) 274.5 ± 62.4	*0.729*
ALT (SGPT) [U/L]	*Median* *IQR*	16.5 (12-19.8)	25.5 (17.5–38.5)	< 0.001*
AST (SGOT) [U/L]	*Median* *IQR*	15 (14–17)	23.5 (18-34.8)	< 0.001*
Bilirubin (mg/dL)	*Median* *IQR*	0.4 (0.3–0.5)	0.4 (0.3–0.6)	*0.053*

Independent Samples T test for parametric quantitative data between the two groups

Mann Whitney test for non-parametric quantitative data between the two groups

*: Significant level at P value less than 0.05

**Table 3:** Fibrosis scores and steatosis staging between control and MAFLD group

		Control	MAFLD	*P* value
		*N* = 60	*N* = 60	
FIB4 score	*Median* *IQR*	0.2 (0.2–0.4)	0.6 (0.5–1.1)	< 0.001*
APRI score	*Median* *IQR*	0.1 (0.1–0.2)	0.2 (0.2–0.3)	< 0.001*
NAFLD score	*Median* *IQR*	-1.5 (-1.8–0.9)	-0.1 (-0.8-0.5)	< 0.001*
Shear wave	*Range* *Mean ± SD*	(3.1–4.4) 4 ± 0.3	(3.6–10.6) 6.5 ± 1.5	< 0.001*
Fibrosis stage	*F0* *F1*	60(100%) 0(0%)	44(73.3%) 16(26.7%)	< 0.001*
Steatosis	*No* *Grade I* *Grade II*	60(100%) 0(0%) 0(0%)	0(0%) 24(40%) 36(60%)	< 0.001*

**Table 4:** Relation between serum VIT D, Fibrosis scores and steatosis staging in the studied groups

		Normal VIT. D	VIT. D deficiency	*P* value
		*N* = 78	*N* = 42	
FIB4 score	*Median* *IQR*	0.3 (0.2–0.5)	0.5 (0.4–0.8)	< 0.001*
APRI score	*Median* *IQR*	0.2 (0.1–0.2)	0.2 (0.1–0.3)	0.034*
NAFLD score	*Median* *IQR*	-1.2 (-1.7–0.4)	-0.3 (-1.4-0.5)	0.002*
Shear wave	*Range* *Mean ± SD*	(3.1–10.6) 4.8 ± 1.5	(3.5–10.6) 5.9 ± 1.8	< 0.001*
Fibrosis stage	*F0* *F1*	71(91%) 7(9%)	33(78.6%) 9(21.4%)	*0.056*
Steatosis	*No* *Grade I* *Grade II*	51(65.4%) 12(15.4%) 15(19.2%)	9(21.4%) 12(28.6%) 21(50%)	< 0.001*

**Table 5:** Correlation between HOMA IR and VIT D in the studied groups

	HOMA IR					
	All group		Control		MAFLD	
	*r*	*P* value	*r*	*P* value	*r*	*P* value
VIT D	***-0.314***	< 0.001*	*0.247*	*0.057*	*-0.126*	*0.336*
Body Weight (kg)	***0.344***	< 0.001*	***0.323***	0.012*	*-0.054*	*0.683*
Height (cm)	*-0.014*	*0.877*	***0.364***	0.004*	*-0.080*	*0.541*
Body Mass Index	***0.347***	< 0.001*	*-0.027*	*0.839*	*0.001*	*0.996*
Waist Circumference (cm)	***0.332***	< 0.001*	*0.225*	*0.084*	*-0.124*	*0.346*
Age (year)	***0.204***	0.025*	*-0.146*	*0.267*	*-0.135*	*0.303*
HBA1c	***0.363***	< 0.001*	***0.409***	0.001*	*0.033*	*0.804*
Plasma Glucose (R) [mg/dL]	*0.081*	*0.380*	***-0.342***	0.007*	*0.155*	*0.238*
ALT (SGPT)	***0.221***	0.015*	*0.123*	*0.349*	*0.037*	*0.781*
AST (SGOT)	***0.228***	0.012*	*0.248*	*0.056*	*0.025*	*0.851*
Bilirubin (Total)	*0.013*	*0.888*	*-0.154*	*0.241*	*-0.102*	*0.439*
Bilirubin (Direct)	*0.091*	*0.322*	*-0.098*	*0.456*	*-0.015*	*0.911*
WBCs	*0.060*	*0.514*	*-0.092*	*0.485*	*0.104*	*0.427*
Hemoglobin	*0.063*	*0.495*	***-0.275***	0.033*	*-0.163*	*0.214*
Platelet Count	*-0.050*	*0.585*	*-0.187*	*0.154*	*-0.026*	*0.841*
Cholesterol	***0.200***	0.029*	*-0.074*	*0.574*	*-0.024*	*0.855*
Triglyceride	***0.247***	0.006*	***0.335***	0.009*	*0.077*	*0.559*
HDL	*-0.144*	*0.116*	*-0.205*	*0.115*	*-0.073*	*0.581*
LDL	*0.165*	*0.071*	*-0.108*	*0.413*	*-0.017*	*0.896*
CRP	*0.121*	*0.189*	*0.143*	*0.274*	*-0.019*	*0.888*
INSULIN	***0.868***	< 0.001*	***0.948***	< 0.001*	***0.843***	< 0.001*
FIB4 score	***0.185***	0.044*	*0.043*	*0.744*	*-0.123*	*0.351*
APRI score	***0.234***	0.010*	*0.166*	*0.204*	*0.089*	*0.497*
NAFLD score	*0.081*	*0.376*	*0.102*	*0.439*	*0.005*	*0.971*
Shear wave	***0.344***	< 0.001*	*-0.206*	*0.114*	*0.049*	*0.711*
Fibrosis stage	***0.206***	0.024*	***----***	*----*	*0.012*	*0.928*
Steatosis stage	***0.435***	< 0.001*	*----*	*----*	*0.036*	*0.783*

**Table 6:** Logistic regression analysis for prediction of insulin resistance among all participants from both control and NAFLD groups combined

	Simple logistic regression			Multiple logistic regression			Multiple stepwise logistic regression		
	OR	95% CI	*P* value	AOR	95% CI	*P* value	OR	95% CI	*P* value
Shear wave	**1.7**	**1.3–2.2**	< 0.001*	1.31	0.9–1.9	0.174			
Vitamin D Normal Deficiency	**Ref.** **3.4**	**1.4–8.3**	0.008*	Ref. 1.26	0.4–4.04	0.693			
BMI	**1.2**	**1.1–1.4**	< 0.001*	1.07	0.9–1.3	0.397			
WC	**1.08**	**1.04–1.1**	< 0.001*	**1.06**	**1.01–1.12**	0.017*	**1.07**	**1.04–1.11**	< 0.001*
Age	**1.04**	**1.01–1.08**	0.025*	**0.94**	**0.88–0.99**	0.048*			
TG	**1.01**	**1.004–1.02**	0.004*	1.006	0.99–1.01	0.172	1.008	1-1.02	0.061
LDL	**1.02**	**1.004–1.03**	0.005*	1.01	0.99–1.02	0.141			

OR: Odds Ratio

AOR: Adjusted Odds Ratio

CI: Confidence interval

NA: Not Applicable

## Data Availability

No datasets were generated or analysed during the current study.

## Abbreviations

VIT D: Vitamin D
HOMA: IR-Homa insulin resistance
NAFLD: Nonalcoholic associated fatty liver disease
MAFLD: Metabolic associated fatty liver disease
BMI: Body mass index
NFS: NAFLD Fibrosis Score
APRI: AST to Platelet ratio
